# Society Meetings

**Published:** 1916-04

**Authors:** 


### SOCIETY MEETINGS

**Brief reports and announcements of meetings of societies of Western New York, and those of general scope, are requested from Secretaries. Copy should be on hand the fifteenth of the month. Full transactions will be published at cost of composition.**

---

**DOCTOR: Is your Society properly represented here? If not, please bring our standing announcement to the attention of the members.**

**The Alienists and Neurologists of the U. S.** will hold their fifth annual meeting in Chicago, June 19-23, under the auspices of the Chicago Medical Society. A general invitation is extended to the medical profession. Feeblemindedness, with special reference to legislation, will be considered.

---

**The Columbia Alumni Assn.** held its annual dinner at the Ellicott Club, Buffalo, Mch. 13. The principal speech was by Dr. Walton Martin, Prof. of Clinical Surgery, describing his experiences in the military branch of the American Hospital in the war zone.

---

**The Rochester Academy of Medicine,** Section III, met Mch.

8. Dr. Jas. K. Quigley gave a paper on Vomiting of Pregnancy; Dr. Clyde C. Sutter on Diet during Pregnancy and the Puerperium.

---

**The Elmira Academy of Medicine** held its regular monthly meeting Mch. 1. Dr. W. A. Dewitt of Blossburg, Pa., read a paper on Local Anaesthesia; Dr. A. H. Baker of Elmira on "Should Auld Acquaintance Be Forgot?"

---

**The Buffalo Academy of Medicine** has held the following meetings since our last report: Mch. 1, Surgical Section, Transplantation of Fascia and Bone, with stereopticon views, Dr. J. T. Rugh of Philadelphia.

Mch. 8, Medical Section, Modern Development of Physical and Colloid Chemistry, G. H. A. Clowes, Ph. D.; Symposium on the Diatheses in Medicine: Internal Medicine, Dr. A. E. Woehnert; Surgery, Dr. H. A. Smith; Ear, Nose and Throat, Dr. J. F. Fairbairn; Paediatrics, Dr. J. A. Ragone; Nerves, Dr. L. Kauffman.

Mch. 15, Section of Obstetrics and Gynaecology, Spontaneous Rupture of Uterus, Dr. F. C. Goldsborough; Premature Separation of Placenta, Dr. E. A. D. Clarke; Haemorrhage into Adrenals, Dr. W. F. Jacobs; Pituitrin in Obstetrics, Haemorrhage in New-born, Dr. N. Kavinoky; Eclampsia, Dr. Wm. M. Brown of Rochester.

Mch. 22, Stated Meeting under auspices of Section of Pathology; Relation of Laboratory Worker to Practitioner, Dr. A. A. Thibaudeau; Sudden Death, with lantern slides, Dr. H. U. Williams.

Nominations were made for the election in June, as follows: President, Dr. Lawrence Hendee; Treasurer, Dr. B. F. Schreiner; Trustee, Dr. W. W. Plummer.

---

**The New York State Homoeopathic Medical Society** will hold its annual meeting in Rochester, April 11-12, under the presidency of Dr. J. S. Dowling.

---

**The Medical Society of the County of Erie,** held its postponed meeting Monday, February 28, 1916, at 8:30 p. m., in Townsend Hall, 25 Niagara Square.

The Secretary read the minutes of the Annual Meeting held December 20th, 1915, and also the minutes of the Council meetings held January 13th, 1916, and February 14th, 1916, which were approved.

Applications for admission to the Society were received from:

Dr. Harry J. Hammond, 1058 Elk St., Buffalo, N. Y.; graduate of the University of Michigan, 1909; graduate in medicine from the Medical Department of the University of Cincinnati, June 14th, 1913; also a member of the Ohio State Society and the Academy of Medicine of Cincinnati. Dr. Charles F. Dowitz, 436 Franklin St., Buffalo; graduate of the University of Buffalo, June 6th, 1914. Dr. Evelyn Hollis Hunt, 219 Bryant St., Buffalo; residence, Hamburg, N. Y.; graduate of the University of Buffalo, June, 1915. Each of these applicants were separately voted upon and admitted to membership.

Dr. A. T. Lytle, submitted the following amendments to the By-laws:

That Chapter IX of the By-laws be so amended that Section 4 shall become Section 2; that Section 2 become Section 3; that Section 3 be abolished and that new Sections 4, 5 and 6 shall be created as follows:

Sec. 4—Nominations for elective offices may be made at the regular meeting next preceding the annual meeting; nominations so made shall be printed in the notice of the annual meeting.

Sec. 5—For use at the annual election the Secretary shall prepare printed ballots which shall contain under the proper titles the names of those nominated according to Chapter IX, Section 4, and also space in which may be written the name of any other member for whom a member desires to vote.

Sec. 6—Voting shall only be done by members in person depositing a ballot with tellers who shall be appointed by the President and who shall report the result of the election to the Society before the adjournment of the annual meeting. The polls shall remain open for not less than two hours after the opening of the annual meeting.

(Signed) ALBERT T. LYTLE.

These amendments were laid on the table in conformity with the By-laws. There being no further business the following Scientific Program was proceded with:

Program. Business Session. Symposium on "Public School Education, from the Standpoint of the Physician." Is the school doing its full duty by the child? Dr. H. H. Glosser. Education and Mental Preparedness, Dr. F. Park Lewis. Education and Physical Preparedness, Dr. James W. Putnam. Medical Inspection—its Results, Dr. De Lancey Rochester. Discussion: Dr. Thomas H. McKee, Hon. Adelbert Moot, Supt. Henry P. Emerson, Miss Mary H. Lewis, Principal A. Duschak, Principal A. G. Bugbee. General discussion.

